# Endocrine effect of ghrelin on histological, hormonal and morphometric parameters of the pituitary gland and the possibility of its clinical application

**DOI:** 10.5937/jomb0-61972

**Published:** 2026-01-28

**Authors:** Jovana Čukuranović-Kokoris, Verica Milošević, Darko Stevanović

**Affiliations:** 1 University of Nis, Faculty of Medicine, Department of Anatomy, Nis; 2 Center for Life Science, Beth Israel Deaconess Medical Centre, Harvard Medical School, USA

**Keywords:** ghrelin, pituitary cells, hormonal status, morphometry, clinical application, grelin, celije hipofize, hormonski status, morfometrija, klinička primena

## Abstract

**Background:**

Ghrelin, a brain-intestinal hormone, is a growth hormone (GH) secretagogue. Because it regulates appetite and is secreted most prominently before meals, it is often described as a "hunger hormone". It consists of 28 amino acids. Given the connection between nutritional status and energy metabolism, it suggests that disorders in these areas can lead to anorexia, especially during aging. This review article aims to demonstrate ghrelin's influence on the histological, hormonal, and stereological characteristics of pituitary cells, as well as its potential clinical applications.

**Methods:**

To write this review, we performed an electronic literature search through Google Scholar and PubMed databases with the terms ghrelin, structure, pituitary cells, metabolism, and aging, with reference to the authors and co-authors of published works related to this topic, as well as the option "related articles", which were associated with the content of this publication.

**Results:**

The topic of this review article relates to the structure, morphometric and hormonal characteristics of adrenocorticotropic (ACTH), somatotropic (GH), and gonadotropic (FSH and LH) pituitary cells in control and ghrelin-treated rats.

**Conclusions:**

This review showed that central administration of nanomolar doses of ghrelin in rats modulates the immunohistomorphometric and hormonal characteristics of pituitary hormone-producing cells. The changes are particularly significant in the volume of corticotropes, somatotropes, and luteinising hormone (LH)-producing cells, their volume density, and the levels of hormones they secrete, compared with the control group.

## Abstract (sr)

**Uvod:**

Grelin, moždano-crevni hormon, je sekretagog hormona rasta (GH), a pošto reguliše apetit i najviše se luči pre obroka, opisuje se kao "hormon gladi". Sastoji se od 28 aminokiselina. S obzirom na vezu između nutritivnog statusa i energetskog metabolizma, sugeriše se da poremecaji u ovim oblastima mogu dovesti do anoreksije, posebno tokom starenja. Cilj ovog preglednog članka je da pokaže uticaj grelina na histološke, hormonske i stereološke karakteristike celija hipofize, kao i mogucnost njegove kliničke primene.

**Metode:**

Da bismo napisali ovaj pregled, izvršili smo elektronsku pretragu literature putem baza podataka "Google Scholar PubMed" i sa terminima grelin, struktura, celije hipofize, metabolizam, starenje sa pozivanjem na autore i koautore objavljenih radova vezanih za ovu temu, kao i opciju "povezani članci", koji su bili povezani sa sadržajem ove publikacije.

**Rezultati:**

Tema ovog preglednog članka odnosi se na strukturu, morfometrijske i hormonske karakteristike adrenokortikotropnih (ACTH), somatotropnih (GH), gonadotropnih (FSH i LH) celija hipofize kod kontrolnih i pacova tretiranih grelinom.

**Zaključak:**

Ovaj pregled je pokazao da centralna primena nanomolarnih doza grelina pacovima ima modulatornu ulogu na imunohistomorfometrijske i hormonske karakteristike celija hipofize koje proizvode hormone. Promene su posebno značajne u zapremini kortikotropa, somatotropa i celija koje proizvode luteinizirajuci hormon (LH), njihovoj zapreminskoj gustini i nivoima hormona koje luče u poređenju sa kontrolnom grupom.

## Introduction

**Figure 1:**
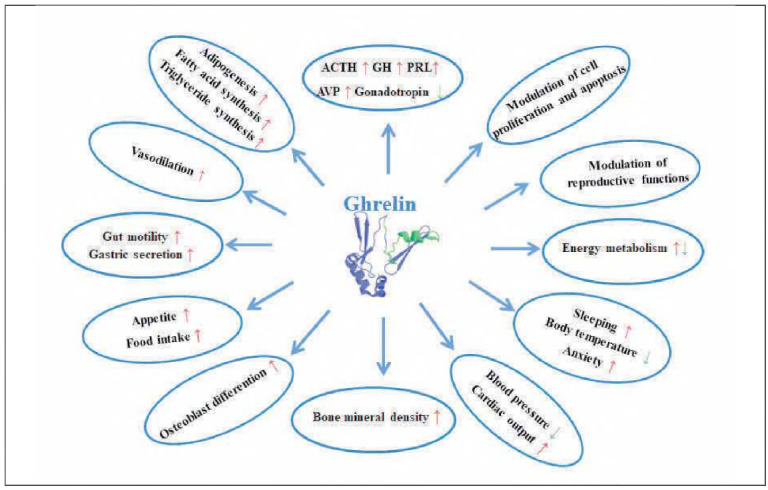
The various physiological functions of ghrelin in the body: ↑increase, ↓decrease; ACTH - adrenocorticotropic hormone; GH - growth hormone; PRL - prolactin; AVP - arginine-vasopressin.

The hormone ghrelin is an orexigenic hormone that was purified from the stomach more than 20 years ago and described by Koima et al. [1]. This hormone stimulates appetite [2], promotes gastric acid secretion and motility, facilitates lipogenesis, inhibits lipid oxidation, utilises carbohydrates as a fuel source, and conserves fat [3]. It is secreted before meals, which is why it is called the »hunger hormone« [4]. The human ghrelin gene is located on chromosome 3 (3p25-26) [5], spans six exons, and is approximately 7.2 kb in length. To form the mature mRNA that is translated into pre-proghrelin, which consists of 117 amino acids, the ghrelin gene undergoes transcription [6]. In the endoplasmic reticulum, pre-proghrelin is cleaved by proteases to form proghrelin, which contains 94 amino acids [6]. In the subsequent biochemical process, proghrelin is converted to mature ghrelin [7], which is then converted to des-acyl ghrelin and acyl ghrelin by ghrelin-O-acyltransferase [8]. Mature ghrelin contains 28 amino acids [7]. It is assumed that acylated ghrelin (AG) is converted to obestatin [9]. More than 90% of desacyl ghrelin and less than 10% of AC circulate in the circulation [10]. It is known that the acyl group of ghrelin is responsible for binding ghrelin to the growth hormone secretagogue receptor (GHS-R) [1]. The primary function of AG is the secretion of growth hormone (GH), and the function of diacylglycerol (DAG) is lipogenesis [11]. Ghrelin has multiple functions, including physiological, biological and pathological roles in vertebrates [12]. In addition to humans, it has been identified in many animal species, including frogs, fish, chickens, pigs, mice, and rats [13]. Human ghrelin differs from rat ghrelin by only two amino acids at positions 11 and 12 [1] [14]. It has been observed that after surgical removal of the gastric mucosa, C-ghrelin concentration decreases in both humans [10] and rats (approximately 80%) [15]. This hormone is widely distributed across various tissues, including the hypothalamus, pituitary gland, kidneys, heart, intestines, pancreas, adrenal gland, and testes [9], indicating its importance in both endocrine and paracrine roles [16]. Ghrelin is involved in a wide range of biological processes; it is crucial for memory, learning, sleep, cognition, and the senses of smell and taste [2], as well as the maintenance of energy and bone homeostasis. It has anti-atrophic and cardioprotective functions [17]. Its importance in stimulating skeletal muscle regeneration after injury has been observed [2]. As a »hunger hormone«, ghrelin stimulates appetite and food intake after binding to receptors and thereby activating orexigenic neurons in the arcuate nucleus of the hypothalamus [17]. Numerous studies have shown that aging is associated with a decrease in appetite, leading to a corresponding decline in energy intake of approximately 1% per year [18] [19]. During this process, metabolic pathways are disrupted, leading to pathological weight loss and malnutrition [18]. The physiological functions of ghrelin are shown in Figure 1.

Research by Landi et al. [20] showed that as people age, ghrelin concentrations decrease, indicating an adverse effect on appetite and food intake, which should be an additional incentive for scientists to investigate further its potential for safe clinical application in »anorexia of aging«. Research has shown that ghrelin has therapeutic implications, as it induces autophagy, and its inhibitory effects protect the body from unwanted inflammation [21].

### Histological characteristics of pituitary cells after ghrelin treatment

**Figure 2:**
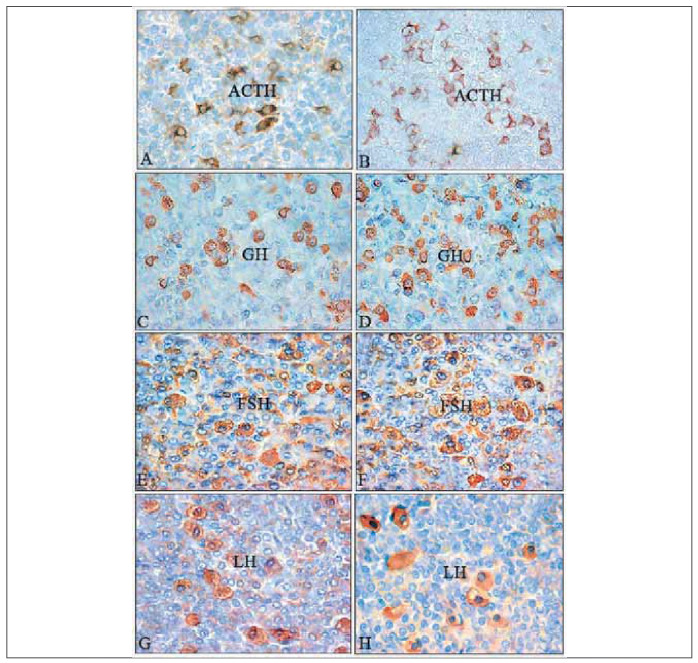
Representative micrographs of immunopositive cells in the pars distalis of the pituitary gland from control: A, C, E, G (ACTH, GH, FSH, LH respectively); from ghrelin treated rats: B, D, F H (ACTH, GH, FSH, LH respectively). ACTH-corticotropic cells, GH-somatotropic cells, follicle-stimulating-FSH-producing cells, luteinizing-LH-producing cells. Peroxidase-antiperoxidase (PAP) method.

Adrenocorticotropic (ACTH) cells in the control pituitary of male rats are mainly found in small groups near the capillaries. They are irregularly shaped, mostly stellate, and are located in the central part of the pars distalis. These cells are intensely stained, with clearly visible nuclei surrounded by immunopositive, granular cytoplasm (Figure 2A) [22] [23]. In male cadavers, immunohistochemically positive ACTH cells are darkly stained, with voluminous cytoplasm and an eccentric nucleus, and they typically appear starshaped, oval, or polygonal [24] [25]. In treated animals with ghrelin, neither the localisation nor the shape of ACTH-immunoreactive cells was altered compared with the control (Figure 2B). Secretory granules in these animals are more numerous, darker in colour than in controls, and distributed along the plasma membrane (Figure 2B) [22].

Histological analysis of ACTH-immunopositive cells in high-fed (HF) and food-restricted (FR) rats revealed no significant changes in shape or position. Still, they appeared darker and smaller than in normal-fed (NF) controls. Ghrelin treatment did not alter the shape of ACTH cells in HF and FR rats compared to their respective controls; however, they became more lightly stained and larger, with granules distributed around the periphery of the cytoplasm [26].

In control male rats, somatotropic (GH) cells are immunocytochemically positive for GH and are ovoidal or pyramidal, with a centrally located spherical nucleus. Intensely stained GH cells were usually located along the sinusoid (Figure 2C) [27] [28]. Pituitary immunoreactive GH cells of the adenohypophysis in a male cadaver were polygonal with a nucleus that is euchromatic and eccentrically placed [29] [30]. In rats treated with ghrelin, immunopositive GH cells were more numerous and larger, and their localisation and shape were generally not significantly altered compared to controls. Darker, more numerous, and smaller secretory granules were distributed mainly at the periphery of the cytoplasm (Figure 2D) [27].

Thyrotropic (TSH) cells in adult control rats were found in the distal part of the pituitary gland, mostly singly or localised in small groups, and immunoreactivity was not found in the cytoplasm. In rats administered ghrelin centrally, TSH cells were often degranulated in the peripheral cytoplasm, reduced in size by 13%, and the relative volume density of these cells per unit volume of the pituitary was reduced by 18% compared to controls [31].

Gonadotropic cells (cells that produce follicle-stimulating hormone-FSH and luteinising hormone-LH) were intensely stained (Figure 2E, G). These cells in adult male rats were oval in shape, in contact with capillaries, and distributed singly or in groups in the distal part of the pituitary gland. In both cell types, the nucleus was eccentrically positioned (Figure 2E, G) [32]. In cadaveric men, LH cells are most often polygonal or oval, with numerous granules in the darkly stained cytoplasm [33] [34]. In ghrelin-treated rats, histological analysis revealed that FSH and LH cells did not exhibit significant changes in shape or distribution within the rat pituitary. Both cell types were slightly lighter in colour than controls (Figure 2F, H) [32].

### Hormonal status of pituitary cells after ghrelin treatment

**Figure 3:**
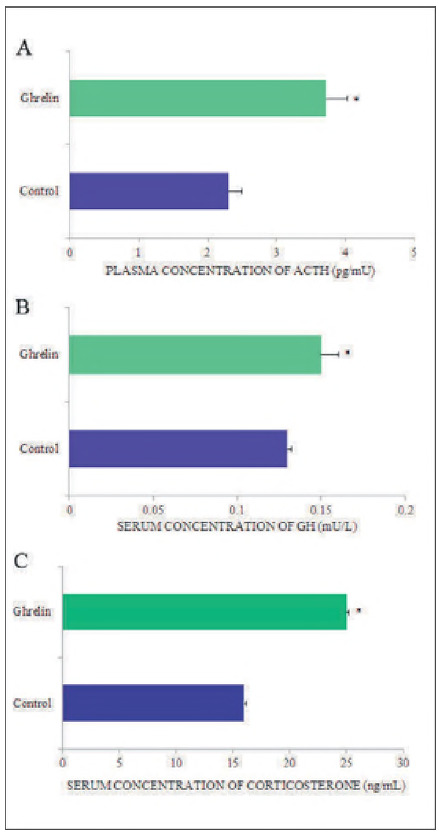
Graphic representation of the concentration of hormone in the blood of control and ghrelin-treated adult rats. All values are expressed as means ± S.D.; *p < 0.05 vs. control; n = 10.

Previous studies have shown that ghrelin, in addition to its strong influence on growth hormone release, plays a crucial role in regulating prolactin (PRL), adrenocorticotropic hormone (ACTH), and cortisol secretion [2] [16] [17]. Studies by Yanagi et al. [21] have shown that ghrelin is involved in stress regulation, and Stevanović et al. [22] found that nanomolar doses of ghrelin significantly increased blood ACTH and corticosterone concentrations (Figure 3A, C) by 62% and 66%, respectively, compared to control rats. In rats fed different diets, it was shown that ACTH and corticosterone levels increased in NF and FR rats, whereas in HF rats, these parameters did not change significantly [26].

Ghrelin in vivo has a stimulatory effect on the hypothalamic-pituitary-adrenal (HPA) axis [35] and on ACTH release, due to the synergistic action of corticotropin-releasing hormone (CRH) and arginine vasopressin (AVP) from the hypothalamus [36]. Centrally administered ghrelin stimulates the HPA axis by releasing both CRH and AVP, whereas systemic ghrelin does not [37]. Increased plasma ghrelin concentration increases serum cortisol levels, independent of increases in central ACTH [38], as the adrenal gland contains ghrelin receptors [39].

Growth hormone (GH) release is regulated by hypothalamic ghrelin and pituitary ghrelin, which, in this case, may have autocrine and paracrine effects [40]. Previous work has shown that the synergistic action of hypothalamic GH-releasing hormone (GHRH) and ghrelin plays an important role in GH secretion from the pituitary gland [41] [42]. Expression of growth hormone secretagogue receptor (GHS-R) mRNA in normal human pituitary, as well as in various pituitary tumours, has been demonstrated [40]. Ghrelin modulates GH secretion by binding to GHS-R1A [43], allowing it to cross the blood-brain barrier [44]. Earlier studies have shown that the effects of ghrelin on GH release in vivo [43] are significantly more potent than those in vitro [1] and more pronounced in humans than in animals [43]. Nanomolar doses of centrally administered ghrelin significantly increase circulating serum GH levels by 15% (Figure 3B) [27] and reduce thyroid-stimulating hormone (TSH) by about 14% [31] compared to corresponding control values. It is hypothesised that by modulating the GH-insulin growth factor axis, desacyl ghrelin may induce GH secretion [45]. An earlier study showed that ghrelin stimulates GH release in vitro but has no significant effect on TSH, prolactin, ACTH, luteinising hormone (LH), or follicle-stimulating hormone (FSH) release [40].

### Morphometric characteristics after ghrelin treatment

**Figure 4:**
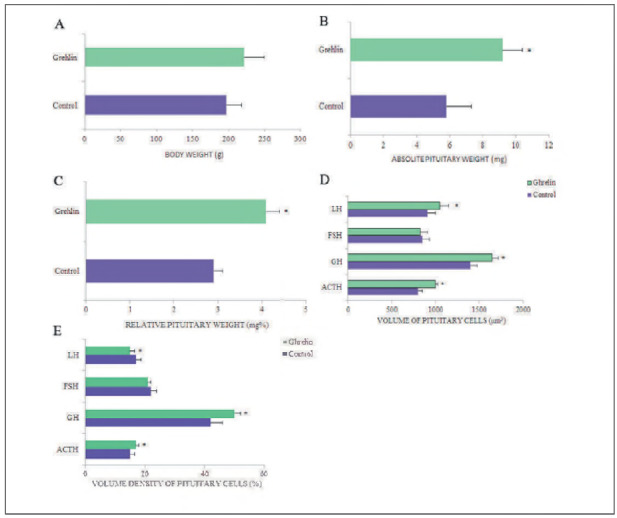
Graphic representation of morphometric parameters: A - body weight (g), absolute - B (mg) and C- relative (mg%) pituitary weights, D - volume of immunopositive ACTH, GH, FSH and LH cells (μm^3^) and E - volume density (%) of immunopositive ACTH, GH, FSH and LH cells in adult control and ghrelin treated rats. ACTH-corticotropic cells, GH-somatotropic cells, follicle-stimulating FSH-producing cells, and luteinizing-LH-producing cells. The values are expressed as means ± SD; *p<0.05 vs. control; n=10.

The body weight of control and ghrelin-treated rats is summarised in Figure 4A. It is clearly seen that body weight increased in adult rats, but this increase was not statistically significant [22] [27] [46]. A substantial increase in body weight was observed after ghrelin administration in peripubertal rats [18]. Previous studies have shown that ghrelin influences food intake [37], weight gain and obesity via homeostatic pathways in the brainstem [47] and hypothalamus [48]. The results of studies on the effects of central administration of nanomolar doses of ghrelin on body weight, food intake, fat deposition, and lipid metabolism during aging (in peripubertal, adult, and middle-aged rats) showed that all these parameters were significantly increased compared to controls [18]. The increase in body weight is most pronounced in the youngest rats and decreases with age, indicating that these parameters are age-dependent [46] [49]. Greater sensitivity to the stimulatory effects of adipogenesis, HPA axis activity, and lipid metabolites has been observed in middle-aged rats after ghrelin treatment [18] [35].

Morphometric characteristics after centrally administered ghrelin on absolute and relative pituitary weight, absolute volume and volume density of ACTH, GH, FSH and LH cells are shown in Figure 4B-E. Absolute and relative pituitary weights were significantly increased in ghrelin-treated animals by 58% and 41%, respectively, compared with the control group (Figure 4B, C) [22] [27].

The absolute weight of the pituitary gland in control FR rats was statistically significantly lower than in control rats with a standard diet [26]. In HF control animals, absolute pituitary weight was 35.9% significantly higher compared to NF control rats. However, ghrelin administration did not cause significant changes in absolute or relative pituitary weight in any of the treated groups compared to the corresponding control [26].

The analysed stereological parameters (volume and volume density) for rat pituitary cells are shown in Figure 4D, E. It can be observed that in adult rats treated with ghrelin, the volume of both ACTH cells and GH cells statistically significantly increased by 17%, and that of LH cells by 7% compared to the corresponding control (Figure 4D) [22] [27] [32], while the volume of FSH cells remained unchanged [32].

The volume density of ACTH and GH cells was significantly increased by 13% and 19%, respectively, in ghrelin-treated rats, and in LH cells this parameter was significantly decreased by 38.9% compared to the control group (Figure 4E) [22] [27] [32]. The volume density of FSH cells was slightly lower than in the control group [32].

Stereological analysis showed that repeated nanomolar doses of ghrelin statistically significantly increased the volume of ACTH cells in NF, HF, and FR rats compared with their corresponding controls. The volume density of these cells in NF, HF, and FR rats was significantly altered only compared with NF controls [26].

### Potential therapeutic capacities of ghrelin

Appetite regulation is very complex (stimulation/inhibition) and includes, in addition to ghrelin, gut peptides, insulin, leptin and the arcuate nucleus of the hypothalamus [50]. Decreased or lost appetite and/or food intake in older adults, described as »anorexia of aging«, was first named and described by Morley et al. [51]. This condition in older adults is associated with chronic diseases, neurodegenerative conditions, and the use of numerous medications. It has recently been recognised as a geriatric syndrome [20]. Loss of appetite and weight in older people may be associated with worsening mental health, including depression and cognitive impairment [52]. It is also believed that dysregulation of the release, action, and/or resistance of peripheral hormones contributes to »anorexia of aging« [53]. Inhibition of appetite is also aided by satiety hormones, including leptin, glucagon-like peptide-1, peptide YY and cholecystokinin, through the involvement of the hypothalamus [10]. It is essential to emphasise that healthy longevity should be a priority for every country; therefore, the timely identification of »anorexia of aging« during preventive health screenings is crucial. Appetite plays a key role in managing the risks associated with aging, to avoid the occurrence of sarcopenia, cachexia, frailty, and disability [54]. Most studies on acylated ghrelin focus on its physiological and pathophysiological effects in the body [8] [21] [55]. In recent years, ghrelin has been shown to play a significant biological role in autophagy, apoptosis, and appetite regulation [8]. In the gastrointestinal tract, autophagy plays a crucial role in the pathogenesis of Crohn's disease, Helicobacter pylori infection, chronic gastritis, various infectious diseases, and gastrointestinal tract cancers [55]. Studies by Ezquerro et al. [56] have demonstrated that ghrelin promotes autophagy by activating adenosine monophosphate-activated protein kinase, thereby influencing glucose and lipid metabolism. Ghrelin, as an anti-inflammatory peptide, acts in inflammatory conditions to prevent cell damage and reduce autophagic flux [57]. Ghrelin analogues have the potential to be used in the treatment of disorders caused by growth hormone deficiency [8]. During development and growth, apoptosis, a regulated process of cell death, plays a crucial role [58]. Depending on the cell type, ghrelin's influence varies, and its role in OE-19 cells of the gastrointestinal tract is particularly noticeable [59].

In patients with sepsis, ghrelin inhibits oxidative stress. It reduces the release of the pro-inflammatory factor high-mobility group box 1 (HMGB1) [60], activates the vagus nerve, and thereby improves the function of the gastrointestinal mucosa [8].

In patients with gastric cancer, a meta-analysis showed significantly lower circulating ghrelin levels compared to healthy individuals [61]. Further research in this field is needed, as there are contradictory results from studies regarding serum ghrelin levels in healthy individuals and those with cancer [62].

Endogenous and exogenous ghrelin have potent beneficial effects on heart failure and hypertrophy, myocardial infarction, as well as on minor arrhythmias and pulmonary hypertension [63]. These effects occur as a result of ghrelin's direct action on cardiac cells as well as modulation of the autonomic nervous system. Ghrelin thus represents a promising new treatment for heart disease compared to other drugs, as it is an endogenous hormone [64].

## Conclusion

The multifunctionality of ghrelin's action and the complexity of regulating the hypothalamic-pituitary axis and target tissues throughout the body are crucial for understanding the diseases and health issues caused by circulating ghrelin deficiency. This review showed that central administration of nanomolar doses of ghrelin in rats modulates the immuno-histomorphometric and hormonal characteristics of pituitary hormone-producing cells. The changes are particularly significant in the volumes, volume densities, and hormone levels of corticotropes and somatotropes compared with controls. The involvement of ghrelin in apoptosis, autophagy, gastrointestinal disorders, colon and gastric cancer, postoperative recovery, and cardiovascular diseases suggests that it is a hormone with multiple effects on health, making it suitable for therapeutic purposes. Ghrelin and its analogues may play a key role in improving post-operative recovery. However, additional research in this field is necessary to ensure patient safety during its use.

## Dodatak

### Acknowledgements

The authors would like to thank the Ministry of Education, Science, and Technological Development of the Republic of Serbia (Grant No. 451-03-137/2025-03/200113) for financial support.

### Conflict of interest statement

All the authors declare that they have no conflict of interest in this work.
